# Prediction of hypertension, hyperglycemia and dyslipidemia from retinal fundus photographs via deep learning: A cross-sectional study of chronic diseases in central China

**DOI:** 10.1371/journal.pone.0233166

**Published:** 2020-05-14

**Authors:** Li Zhang, Mengya Yuan, Zhen An, Xiangmei Zhao, Hui Wu, Haibin Li, Ya Wang, Beibei Sun, Huijun Li, Shibin Ding, Xiang Zeng, Ling Chao, Pan Li, Weidong Wu

**Affiliations:** School of Public Health, Xinxiang Medical University, Xinxiang, Henan Province, China; Indiana University School of Medicine, UNITED STATES

## Abstract

Retinal fundus photography provides a non-invasive approach for identifying early microcirculatory alterations of chronic diseases prior to the onset of overt clinical complications. Here, we developed neural network models to predict hypertension, hyperglycemia, dyslipidemia, and a range of risk factors from retinal fundus images obtained from a cross-sectional study of chronic diseases in rural areas of Xinxiang County, Henan, in central China. 1222 high-quality retinal images and over 50 measurements of anthropometry and biochemical parameters were generated from 625 subjects. The models in this study achieved an area under the ROC curve (AUC) of 0.880 in predicting hyperglycemia, of 0.766 in predicting hypertension, and of 0.703 in predicting dyslipidemia. In addition, these models can predict with AUC>0.7 several blood test erythrocyte parameters, including hematocrit (HCT), mean corpuscular hemoglobin concentration (MCHC), and a cluster of cardiovascular disease (CVD) risk factors. Taken together, deep learning approaches are feasible for predicting hypertension, dyslipidemia, diabetes, and risks of other chronic diseases.

## Introduction

Hypertension, hyperglycemia, and dyslipidemia are disorders defined by dircect mesures of blood pressure, fasting plasma glucose, and triglyceride levels, respectively. These disorders frequently occur with each other and are among the primary risk factors for cardiovascular disease (CVD), the leading cause of morbidity and mortality worldwide [1]. As China faces the ageing of its population, changes in lifestyle and longer life expectancy have led to increased CVD events. CVD now accounts for more than 40% of deaths from all causes [2, 3]. With a rise in CVD on this scale, it is not only a serious public health problem but also a substantial burden on both healthcare systems and budgets. Thus, measures to prevent and control CVD in China are ugently needed.

Over the past few years, advances in the field of digital retinal photography and imaging techniques have made it possible to characterize subtle changes in retinal blood vessels precisely. From retinal fundus images, early microcirculation changes in chronic diseases prior to the onset of obvious clinical complications can be detected directly and non-invasively [4].

Changes in the retina have used by physians to assess a patient’s risk of a number of CVD including diabetes and hypertension [4–7]. That is, these features in the eyes may reflect the conditions of the cardiovascular system. Poplin et al. [8] showed that retinal images alone were sufficient to predict several CVD risk factors such as age, gender, smoking status, blood pressure, and body mass index (BMI). In this study, we predicted hypertension, hyperglycemia, dyslipidemia, and a collection of other risk factors from retinal fundus photographs in a cross-sectional study of chronic diseases in central China using deep learning approaches. The subjects in this study were mainly from rural areas of Xinxiang County, Henan Province, China.

Deep learning is a family of machine learning algorithms based on learning data representations. It allows a machine to be fed raw data and to automatically discover the reprnesentations needed for detection or classification [9, 10]. In recent years, deep learning algorithms such as convolutional neural networks (CNNs) have been widely applied to medical imaging analysis [11–15]. Transfer learning with CNNs is a machine learning technology that learning of a new task (e.g., medical images) relies on the previously learned tasks (e.g., ImageNet, a dataset of millions of common everyday objects), the learning process can be faster, more accurate and need less training data [12]. In recent years, transfer learning has become integral to many applications, especially in medical imaging [12–18]. Many applications on medical imaging have demonstrated promising results and reached expert-level diagnostic accuracies, such as assisting classification of Alzheimer's disease stages using 3D MRI scans [16], detection and quantification of macular fluid in OCT images [17], breast-mass identification using mammography scans [18], diagnosis of pediatric pneumonia using chest X-ray images [12] and detection of diabetic retinopathy in retinal fundus photographs [14].

The aim of the present study was to develop automated artificial intelligence models, applicable to large-scale population screening, which could be used to predict hypertension, hyperglycemia, dyslipidemia, and other risk factors for CVD based on retinal fundus images [8, 19]. Large-scale detection and early treatment of hypertension, hyperglycemia, and dyslipidemia enabled by this technology, especially in rural areas, may reduce both cardiovascular events and the economic burden on national health care systems.

## Materials and methods

### Study population

The dataset in this study was generated from April to June, 2017 through recruiting 625 participants, aged 24–83 years, across several rural villages of Xinxiang County, Henan province in central China to assess the relationships between retinal vascular profiles and chronic diseases. The protocol of this study was reviewed and approved by the Ethics Committee of Xinxiang Medical University for Human Studies (IRB registration number XY-HS04). Each subject signed an informed consent form and went through a series of health measurements and questionnaires. Blood samples of each subject were collected to assess biochemical alterations from April 20 to June 6, 2017.

Trained physicians collected the subjects' blood samples in the morning after overnight fasting using standard methods. Trained and certified medical students measured resting blood pressure using an automated OMRON HEM-7071 professional portable blood pressure monitor with the participant seated. Anthropometric measurements, including height, waistline, and hip circumference, were measured twice with a tape. Body weight was obtained using an automated weight monitor following the manufacturer's instruction. Body weight and the average of the height, waistline, and hip circumference were used to calculate the BMI and waist-hip ratio (WHR).

Smoking, alcohol drinking, and salt intake statuses were obtained using a questionnaire. For smoking and drinking, the participants were asked to self-identify as a current drinker (drinking more than 12 times in the past year) or smoker (having smoking habits in the past six months), former drinker or smoker, or non-drinker or non-smoker. Those who had a drinking or smoking history were then asked for additional details. For the purpose of this study, the population was binarized into those who were current drinkers or smokers and those who were not. For salt intake status, the participants were asked to self-identify whether their eating habits were salty, and the options included four categories (light, general, salty, very salty). The subjects were also classified into two groups, salty and non-salty intake population.

Paired color retinal fundus photographs of the participants were taken using the Canon CR-2 Digital Non-Mydriatic Retinal Camera. For each subject, we selected the paired 2 images, separately from left and right eyes. However, 28 subjects only have one image be selected since the other one is missing or with low quality. Finally, we obtained 1222 images from 625 subjects. Fundus images of this dataset are consistently sized (2736×1824 pixels).

### Risk factors selected to develop classification models

The primary application of the deep neural network models was in the detection of hypertension, hyperglycemia, and dyslipidemia from retinal fundus images. Besides, we aimed to train deep neural network models to predict a variety of risk factors that are related to the development of hypertension, hyperglycemia and dyslipidemia from retinal fundus images, which included age, BMI, WHR, lifestyle data (drinking, smoking and salty taste status) and biochemical parameters from blood samples (hematocrit (HCT), total bilirubin (T-BIL), direct bilirubin (D-BIL), mean corpuscular hemoglobin concentration (MCHC), total cholesterol (TC) and low-density lipoprotein cholesterol (LDL-C)). The subjects and their corresponding retinal images for each diagnosis category of the above three disorders and other risk factors were separately divided into two classes based on their corresponding classification criterion. For example, for variable ‘hypertension’, the subjects whose systolic BP ≥ 130 or diastolic BP ≥ 85 mmHg or treatment of previously diagnosed hypertension are classified into abnormal group and all other subjects are grouped into normal group; for variable ‘smoking’, the subjects were divided into smoking or none-smoking based on their self-reported information. When training models for each factor, only the subjects with corresponding risk factor outcome information and their fundus images were selected. Classification criterion of each above risk factor are available in **S1 Table.**

### Model development

In this study, we used a transfer learning strategy to process retinal images and to develop models having an accurate diagnosis of hypertension, hyperglycemia, dyslipidemia and other related risk factors. For training and testing processes, we used the open source machine learning platform TensorFlow (https://www.tensorflow.org/) [20]. All the experiments were run on a machine learning workstation with an Intel i7-6850K CPU @ 3.60 GHz with 16 GB of RAM memory and 4 NVIDIA GeForce Titanxp GPU card of 12 GB.

The training process of transfer learning includes loading a pre-trained convolutional neural network model and its pre-trained weights, and then retraining the parameters of the fully-connected and softmax layers to classify images [21]. The pre-trained model used in this study was the Inception-v3 image recognition neural network, which was trained with a dataset of 1000 classes and more than a million images of common everyday objects from the original ImageNet database [22, 23]. Though this Inception-v3 model was not developed for medical image recognition, it has been successfully used for classifying medical images base on transfer learning methods [12, 24], which include classification of retinal fundus images [8, 14, 25]. In this study, the convolutional layers from Inception-v3 were frozen and used as fixed feature extractors. Images were first input to the Inception-v3 neural network, which extracts general features from input images and converts the image data into feature vectors. Then a classification part with fully-connected and softmax layers was trained to classify the images and outcome the predicted labels.

We trained models separately for each selected risk factors. When training each model, the whole dataset is retinal images labeled into two classes base on subjects’ corresponding risk factor outcome information and this risk factor’s classification criterion. Then, the whole dataset was randomly divided into three portions: a training dataset (80%), a tuning validation dataset (10%), and a test dataset (10%). The training and tuning validation datasets were used to develop the model, and the test dataset was used to validate the performance of the final model. During the training processes, a back propagation algorithm was used to optimize the network’s internal parameters [22], and L2 regularization technique was used to avoid overfitting [26, 27].

### Image preprocessing and augmentation

All images were resized into a consistent size (800 × 800 pixels) before training. To correct uneven illumination and brightness, and to adjust variations contrast of retinal images, we pre-processed all the images using the subtractive normalization approach (**S1 Fig**). The image normalization formula is as follows:

$$image_{out}=image\times \alpha +image_{gaussian}\times \beta +\gamma ,$$

where *image* is the original image, and *image*_*gaussian*_ is the image processed by Gaussian filter, *α* = 4, *β* = -4 and *γ* = -128.

Training deep neural networks on imbalanced datasets, in which the majority of data instances belong to one class and far fewer instances belong to others, is an important problem as imbalanced datasets exist widely in the real world [28, 29]. Classifiers trained with imbalanced data are often biased towards the majority class and therefore cause higher misclassification rates for the minority class [28]. To overcome this challenge, only hypertension, hyperglycemia, and dyslipidemia and 13 related risk factors with the ratio of its two classes less than 4:1 were trained to obtain the classification model in this study **(S1 Table)**. Minority classes in each variable were oversampled using an augmentation approach until the two classes were equal. Data augmentation was conducted using Augmentor, which was an image augmentation library designed to aid the artificial generation of image data for machine learning [30].

### Statistical analysis

The output of each prediction model is two continuous numbers from 0 to 1, each referring a probability of each diagnostic label, whose sum is 1. For example, in the hypertension prediction model, the results were presented as ‘hypertension: 0.897 and non-hypertension: 0.103’. The final prediction was based on the predicted labels with a higher probability, which meant that the predicted label in the example above was hypertension. For each risk factor, the accuracy of its prediction model was measured by dividing the number of correctly labeled images by the total number of images that are available in this risk factor. ROC curves were used to plot the false positive rate versus the true positive rate of the model in predicting labels of the test images. The AUC was used to evaluate the model performance for classification of each binary risk factor.

## Results

### Study subjects and characteristics used to develop classification models

We obtained 1222 retinal fundus images from 625 subjects from the cross-sectional syidy of chronic diseases dataset of Henan province in central China. The mean age of the subjects was 54.70 ± 11.67 years, and 55.86% of them were self-identified having at least one ‘chronic disease diagnosed by doctor,’ such as hypertension, hyperlipidemia, diabetes, or coronary disease. All characteristics of the subjects are shown in **Table 1**, which includes 50 variables from blood tests or self-reported questionnaires. In this study, to overcome the problem of machine learning generated by imbalanced dataset, only hypertension, hyperglycemia, dyslipidemia and 13 related risk factors with the ratio of its two classes less than 4:1 were trained to obtain the classification model **(S1 Table).**

**Table 1:** Characteristics of 625 subjects in our chronic disease cohort dataset.

Characteristics	Datasets	Characteristics	Datasets	Characteristics	Datasets
Age: mean, years (s. d.)	54.70(11.67), n = 625	Indirect bilirubin: mean, μmol/L (s. d.)	12.59(5.99), n = 623	MO#: mean, 109/L (s. d.)	0.33(0.11), n = 615
Gender (% male)	39.7, n = 625	ALT: mean, U/L (s. d.)	23.33(24.52), n = 624	EO#: mean, 109/L (s. d.)	0.12(0.11), n = 614
Current Smoker: %	41.19%, n = 624	Alkaline phosphatase: mean, U/L (s. d.)	88.67(26.21), n = 624	BASO#: mean, 109/L (s. d.)	0.03(0.02), n = 614
Drinking: %	26.60%, n = 624	AST: mean, U/L (s. d.)	23.16(14.08), n = 624	RBC: mean, 1012/L (s. d.)	4.82(0.47), n = 615
Moderate exercise: %	90.24, n = 625	Creatinine: mean, μmol/L (s. d.)	61.13(12.82), n = 624	Hematocrit: mean, % (s. d.)	44.61(4.69), n = 615
Salty Taste: %	23.40, n = 624	Urea: mean, mmol/L (s. d.)	4.90(1.32), n = 624	MCV: mean, fL (s. d.)	92.64(6.87), n = 616
PSQI: mean, (s. d.)	3.84(2.73), n = 619	Uric acid: mean, μmol/L (s. d.)	280.85(82.18), n = 624	MCH: mean, pg (s. d.)	29.25(2.12), n = 616
Body mass index: mean, kg/m2 (s. d.)	25.59(3.45), n = 624	Total cholesterol: mean, mmol/L (s. d.)	5.32(1.06), n = 624	MCHC: mean, g/L (s. d.)	316.28(17.50), n = 616
Basal metabolism: mean, kcal (s. d.)	1427.96(219.76), n = 624	Triglyceride: mean, mmol/L (s. d.)	1.72(1.26), n = 623	Hemoglobin: mean, g/L (s. d.)	141.09(16.44), n = 615
Waist-hip ratio: mean, (s. d.)	0.89(0.07), n = 624	LDL-C: mean, mmol/L (s. d.)	2.95(0.78), n = 624	RDW-CV: mean, % (s. d.)	13.50(1.33), n = 615
Body fat ratio: mean, % (s. d.)	31.05(6.51), n = 624	HDL-C: mean, mmol/L (s. d.)	1.34(0.34), n = 624	RDW-SD: mean, fL (s. d.)	44.56(4.91), n = 616
Visceral fat index: mean, (s. d.)	10.07(4.40), n = 625	FPG: mean, mmol/L (s. d.)	6.01(1.59), n = 624	MPV: mean, fL (s. d.)	11.05(0.97), n = 615
Current chronic disease: %	55.86, n = 623	Glycated hemoglobin: mean, % (s. d.)	5.61(1.03), n = 612	PLT: mean, 109/L (s. d.)	249.90(64.24), n = 615
Systolic BP: mean, mmHg (s. d.)	133.02(20.02), n = 625	Insulin: mean, μU/L (s. d.)	8.77(7.17), n = 623	Plateletcrit: mean, % (s. d.)	0.27(0.07), n = 613
Diastolic BP: mean, mmHg (s. d.)	84.11(11.42), n = 625	White blood cell count: mean, 109/L (s. d.)	6.06(1.46), n = 615	PDW: mean, % (s. d.)	12.63(2.03), n = 614
Total bilirubin: mean, μmol/L (s. d.)	16.61(7.43), n = 623	NEUT#: mean, 109/L (s. d.)	3.55(1.15), n = 614	P-LCR: mean, % (s. d.)	46.32(2.32), n = 615
Direct bilirubin: mean, μmol/L (s. d.)	4.02(1.63), n = 624	Lymphocyte count: mean, 109/L (s. d.)	2.03(0.57), n = 615		

n is the number of subjects for whom that measurement was available.

Abbreviations: ALT, alanine aminotransferase; AST, aspartate aminotransferase; BASO#, basophil absolute count; BP, blood pressure; EO#, eosinophil absolute count; FPG, Fasting plasma glucose; HDL-C, high-density lipoprotein cholesterol; LDL-C, low-density lipoprotein cholesterol; MCH, mean corpuscular hemoglobin; MCHC, mean corpuscular hemoglobin concentration; MCV, mean corpuscular volume; MO#, monocytes absolute count; MPV, mean platelet volume; NEUT#, neutrophil absolute count; PDW, platelet distribution width; P-LCR, platelet large cell ratio; PLT, platelet concentration; PSQI, pittsburgh sleep quality index; RBC, red blood cell count; RDW-CV, red blood cell distribution width-coefficient of variation; RDW-SD, red blood cell distribution width-standard deviation.

### Model performance in detecting hypertension, hyperglycemia and dyslipidemia

**Fig 1:**
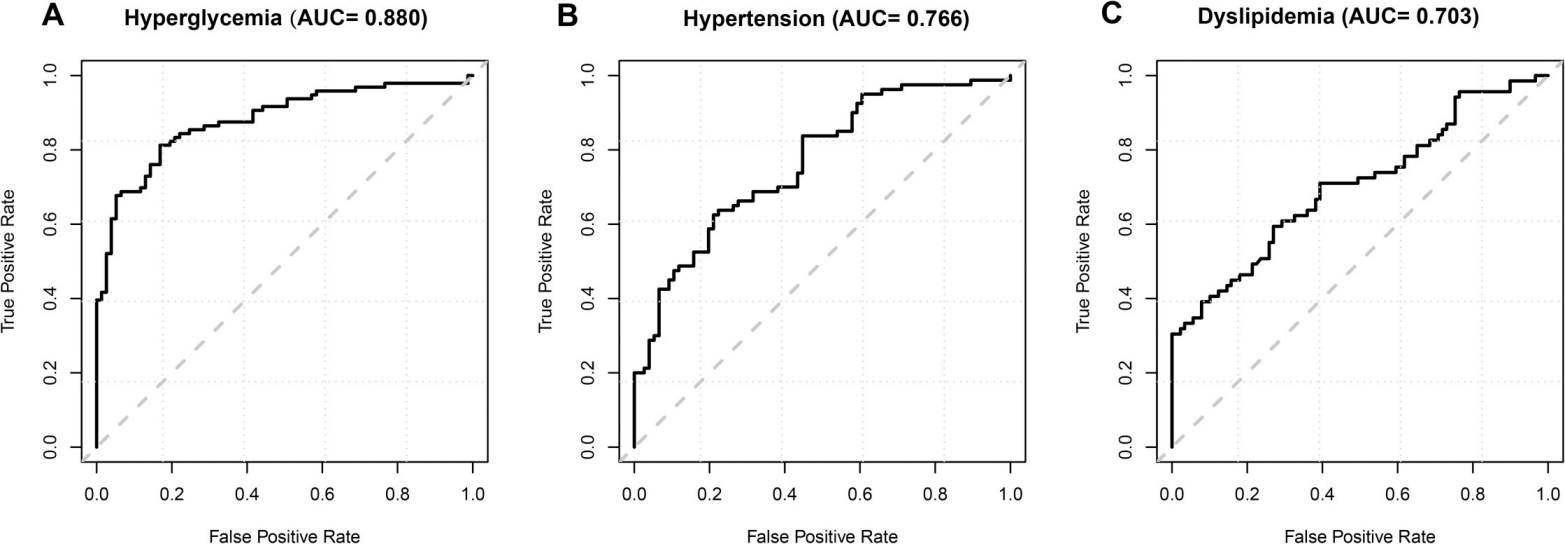
ROC curves for predicted models in detecting three disorders.

We evaluated the models’ performance in detecting hypertension, hyperglycemia and dyslipidemia from retinal fundus images by accessing prediction accuracy and generating the receiver operating characteristic curve (ROC). In the training process, we used an L2 regularization technique to prevent overfitting and stopped the training process when both accuracy and cross-entropy could not be improved further. The accuracy and cross-entropy of the three disorders are shown in **S2 Fig** and the ROC curves are shown in **Fig 1.** As a result, we achieved an accuracy of 78.7% in detecting hyperglycemia, with an area under the ROC curve (AUC) of 0.880; an accuracy of 68.8% in detecting hypertension, with an AUC of 0.766; an accuracy of 66.7% in detecting dyslipidemia, with an AUC of 0.703.

### Model performance in classification of cardiovascular disease risk factors

Although hypertension, hyperglycemia, and dyslipidemia can be discriminated from each other by means of levels of fasting plasma glucose (FPG), systolic blood pressure (SBP) or diastolic blood pressure (DBP), and triglyceride (TG), the diagnosis and prevention of these disorders continues to bother the doctors in clinical practice. The underlying causes of these disorders include genetics, physical inactivity, aging, a proinflammatory state and hormonal changes. Obesity, age, smoking and a collection of CVD risk factors can lead to the development of hypertension, hyperglycemia and dyslipidemia [31, 32]. These risk factors should be considered when attempting to manage the prevention of cardiovascular in clinical practice.

We further selected a number of other parameters that appeared to be related to CVD and trained the deep learning models to classify each parameter via retinal fundus images. Our models achieved an AUC >0.7 in predicting age, gender, drinking status, smoking status, salty taste, BMI, WHR, and HCT **(Table 2).**

**Table 2:** Classification criterion and the model performance of each variable.

Risk factors used for the prediction	Status or cutoff value	Prediction accuracy (%)	AUC
**Hyperglycemia**	**Fasting plasma glucose > 6.1**	**0.787**	**0.880**
**Hypertension**	**Systolic BP > 140 mmHg or Diastolic BP > 90 mmHg**	**0.688**	**0.766**
**Dyslipidemia**	**Triglyceride > 1.71**	**0.667**	**0.703**
Age	>55	0.748	0.850
Gender	Male/Female	0.624	0.704
Drinking	Drinkers/non-drinkers	0.863	0.948
Salty Taste	High-salt diet/lower-salt diet	0.757	0.809
Smoking	Smoker/non-smoker	0.732	0.794
BMI	≦24.0 kg/m^2^	0.712	0.731
WHR	Male<0.9, Female<0.85	0.646	0.704
HCT	Male 40–50%, Female 35–45%	0.698	0.759
MCHC	316–354 g/L	0.605	0.686
T-BIL	3.4–17.1 μmol/L	0.700	0.764
D-BIL	0–3.4 μmol/L	0.650	0.703

**Abbreviation:** BMI, body mass index; BP, blood pressure; D-BIL, direct bilirubin; HCT, hematocrit; MCHC, mean corpuscular hemoglobin concentration; T-BIL, total bilirubin; WHR, waist-hip ratio

## Discussion

Changes in retinal vasculature are associated with cardiovascular disorders such as hypertension, metabolic syndrome, diabetes, and stroke [4, 5, 7]. Long term duration of diabetes and hypertension are the main factors to the onset of some eye diseases, such as diabetic retinopathy (DR) and hypertensive retinopathy (HR) [4]. In recent years, deep learning methods are increasingly used to improve clinical practice by using medical images including retinal fundus images [9, 12, 15]. The performance of these automated models could achieve as accurate as and in some cases superior to human experts in diagnosing diseases [14, 15, 25, 33, 34]. Triwijoyo et al. [33] developed a model of predicting HR, which achieved the prediction accuracy of 0.986. In detecting DR, there were also several studies achieved good performance with AUC > 0.989 [14, 25, 33, 34, 25]. However, these studies were focused on the complications of eyes caused by cardiovascular diseases. For DR, Tapp et al. [35] has shown that the prevalence of DR is less than 10% in those with diabetes duration of less than 5 years. In our study, we generated a retinal fundus image dataset from a population in rural areas of central China, and demonstrate that deep learning models have the ability to predict hypertension (AUC = 0.766), hyperglycemia (AUC = 0.880), and dyslipidemia (AUC = 0.703) using retinal fundus images alone. This result achieved a higher accuracy when comparing with a recent published study by Dai et al. [36], which used a different population in China as well and showed that hypertension can be predicted using fundus images with an accuracy of 0.609. These results demonstrate that early microcirculatory changes may reflect the disorders of some cardiovascular risk factors before the onset of clinical cardiovascular diseases or complication eye diseases. Besides, our study is not limited to predict the above three disorders. Consistent with the study by Poplin et al. [8] in a mainly Caucasian and Hispanic population, we found that cardiovascular risk factors like age, gender, smoking status, and BMI can be predicted directly using retinal fundus images of rural population in central China. Since most of the cardiovascular risk factors can be reflected by retinal fundus images alone, our deep learning methods may, therefore, offer a novel, noninvasive measurement of early changes in the vasculature and allow the identification of people at risk of cardiovascular diseases. Importantly, our results show that applying deep learning to retinal fundus images can also predict blood erythrocyte parameters, including HCT and MCHC **(Table 2)**. Previous studies have confirmed that erythrocyte parameters are associated with cardiovascular diseases, such as metabolic syndrome [37] and that elevated blood erythrocyte parameters can have adverse effects on retinal vessel calibers [38].

CVD, the major cause of death in China, has become a major public health concern [3, 39, 40]. The increasing prevalence of CVD in China is closely linked to a number of risk factors, including hypertension, dyslipidemia, diabetes, smoking, obesity, and metabolic syndrome [31, 39]. An efficient and accurate identification of these risk factors is essential to ensure the prevention and control of CVD. For example, stroke can be reduced by 50% by controlling hypertension [3]. In this study, we applied a deep learning algorithm to analyze retinal fundus images to develop models that, without anthropometry and biochemical data, predicted many cardiovascular risk factors. This technology, coupled with informed policy and intervention strategies, offers a potentially automated approach to preventing and controlling CVD in large populations, especially in rural areas of China.

Despite the good performance of our models, our study has several limitations. Our dataset size is relatively small, although transfer learning algorithm can achieve a highly accurate model with a relatively small training dataset [11–13]. A larger population with more cardiovascular events would make deep learning models that could be trained and evaluated with more accuracy and higher confidence. In addition, employment of the datasets from other sources to validate our models would be beneficial for all these predictions. Overcoming these limitations using these datasets also provides an opportunity to iteratively re-train the deep learning algorithms and improve model performance.

In conclusion, we show that the application of deep learning to retinal fundus images is useful in the prediction of the important CVD risk factors of hypertension, dyslipidemia, diabetes. More importantly, it makes cardiovascular risk assessment of a large population both technically and economically feasible. Our work also suggests that deep learning model analysis of retinal fundus images is useful to diagnose widespread systemic vascular diseases.

## Supporting information

S1 Checklist STROBE statement—checklist of items that should be included in reports of observational studies. (DOCX)

S1 Fig Image pre-processing. A: Original image, B: Image after pre-processing.
(TIF)

S2 Fig Plots showing the model performance in the training and validation datasets. The accuracy of three disorders are shown in A (Hyperglycemia), B (Hypertension) and C (Dyslipidemia). The cross-entropy of the three disorders were shown in D-F. Training dataset: orange; Validation dataset: blue.
(TIF)

S1 Table Group members of subjects and their corresponding retinal images in each variable and their image number ratio of each two groups. (DOCX)
