# Supplementary material for: Prediction of hypertension, hyperglycemia and dyslipidemia from retinal fundus photographs via deep learning: A cross-sectional study of chronic diseases in central China
Source: PLoS One. 2020 May 14;15(5):e0233166. doi: 10.1371/journal.pone.0233166 (PMC7224473; doi:10.1371/journal.pone.0233166)
Supplement: S1 Checklist — (DOCX) [file pone.0233166.s001.docx]

STROBE Statement—checklist of items that should be included in reports of observational studies

|  | Item No. | Recommendation | Page  No. | Relevant text from manuscript |
| --- | --- | --- | --- | --- |
| **Title and abstract** | 1 | (*a*) Indicate the study’s design with a commonly used term in the title or the abstract | 1 | title |
|  |  | (*b*) Provide in the abstract an informative and balanced summary of what was done and what was found | 2 | Abstract |
| Introduction | | | |  |
| Background/rationale | 2 | Explain the scientific background and rationale for the investigation being reported | 3,4 | Changes in the retina have long been used by physians to assess a patient’s risk of a number of CVD risk factors including diabetes, hypertension, age, and smoking [4-7]. That is, these features in the eyes may reflect the conditions of the cardiovascular system. Poplin et al. [8] showed that retinal images alone were sufficient to predict several risk factors such as age, gender, smoking status, blood pressure, and body mass index (BMI). In this study, we predicted hypertension, hyperglycemia, dyslipidemia, and a collection of other risk factors from retinal fundus photographs in a cross-sectional study of chronic diseases in central China using deep learning approaches. The subjects in this study were mainly from rural areas of Xinxiang County, Henan Province, China.  Deep learning is a family of machine learning algorithms based on learning data representations. It allows a machine to be fed raw data and to automatically discover the reprnesentations needed for detection or classification [9, 10]. In recent years, deep learning algorithms such as convolutional neural networks (CNNs) have been widely applied to medical imaging analysis [11-15]. Transfer learning with CNNs is a machine learning technology that learning of a new task (e.g., medical images) relies on the previously learned tasks (e.g., ImageNet, a dataset of millions of common everyday objects), the learning process can be faster, more accurate and need less training data [12]. In recent years, transfer learning has become integral to many applications, especially in medical imaging [12-18]. Many applications on medical imaging have demonstrated promising results and reached expert-level diagnostic accuracies, such as assisting classification of Alzheimer's disease stages using 3D MRI scans [16], detection and quantification of macular fluid in OCT images [17], breast-mass identification using mammography scans [18], diagnosis of pediatric pneumonia using chest X-ray images [12] and detection of diabetic retinopathy in retinal fundus photographs [14]. |
| Objectives | 3 | State specific objectives, including any prespecified hypotheses | 4,5 | The aim of the present study was to develop automated artificial intelligence models, applicable to large-scale population screening, which could be used to predict hypertension, hyperglycemia, dyslipidemia, and other risk factors for CVD based on retinal fundus images [8, 19]. Large-scale detection and early treatment of hypertension, hyperglycemia, and dyslipidemia enabled by this technology, especially in rural areas, may reduce both cardiovascular events and the economic burden on national health care systems. |
| Methods | | | |  |
| Study design | 4 | Present key elements of study design early in the paper | 7,8 | In this study, the convolutional layers from Inception-v3 were frozen and used as fixed feature extractors. Images were first input to the Inception-v3 neural network, which extracts general features from input images and converts the image data into feature vectors. Then a classification part with fully-connected and softmax layers was trained to classify the images and outcome the predicted labels.  We trained models separately for each selected risk factors. When training each model, the whole dataset is retinal images labeled into two classes base on subjects’ corresponding risk factor outcome information and this risk factor’s classification criterion. Then, the whole dataset was randomly divided into three portions: a training dataset (80%), a tuning validation dataset (10%), and a test dataset (10%). The training and tuning validation datasets were used to develop the model, and the test dataset was used to validate the performance of the final model. During the training processes, a back propagation algorithm was used to optimize the network’s internal parameters [22], and L2 regularization technique was used to avoid overfitting [26, 27]. |
| Setting | 5 | Describe the setting, locations, and relevant dates, including periods of recruitment, exposure, follow-up, and data collection | 5 | The dataset in this study was generated from April to June, 2017 through recruiting 625 participants, aged 24-83 years, across several rural villages of Xinxiang County, Henan province in central China to assess the relationships between retinal vascular profiles and chronic diseases. The protocol of this study was reviewed and approved by the Ethics Committee of Xinxiang Medical University for Human Studies (IRB registration number XY-HS04). Each subject signed an informed consent form and went through a series of health measurements and questionnaires. Blood samples of each subject were collected to assess biochemical alterations from April 20 to June 6, 2017. |
| Participants | 6 | (*a*) *Cohort study*—Give the eligibility criteria, and the sources and methods of selection of participants. Describe methods of follow-up  *Case-control study*—Give the eligibility criteria, and the sources and methods of case ascertainment and control selection. Give the rationale for the choice of cases and controls  *Cross-sectional study*—Give the eligibility criteria, and the sources and methods of selection of participants | 5 | The dataset in this study was generated from April to June, 2017 through recruiting 625 participants, aged 24-83 years, across several rural villages of Xinxiang County, Henan province in central China to assess the relationships between retinal vascular profiles and chronic diseases.  The participants were older than 18 years, met the inclusion criteria, and were competent and willing to provide written consent. |
|  |  | (*b*) *Cohort study*—For matched studies, give matching criteria and number of exposed and unexposed  *Case-control study*—For matched studies, give matching criteria and the number of controls per case |  |  |
| Variables | 7 | Clearly define all outcomes, exposures, predictors, potential confounders, and effect modifiers. Give diagnostic criteria, if applicable | 23-25 | Tables 2 and S1 |
| Data sources/ measurement | 8* | For each variable of interest, give sources of data and details of methods of assessment (measurement). Describe comparability of assessment methods if there is more than one group | 5,6 | Trained physicians collected the subjects' blood samples in the morning after overnight fasting using standard methods. Trained and certified medical students measured resting blood pressure using an automated OMRON HEM-7071 professional portable blood pressure monitor with the participant seated. Anthropometric measurements, including height, waistline, and hip circumference, were measured twice with a tape. Body weight was obtained using an automated weight monitor following the manufacturer's instruction. Body weight and the average of the height, waistline, and hip circumference were used to calculate the BMI and waist-hip ratio (WHR).  Smoking, alcohol drinking, and salt intake statuses were obtained using a questionnaire. For smoking and drinking, the participants were asked to self-identify as a current drinker (drinking more than 12 times in the past year) or smoker (having smoking habits in the past six months), former drinker or smoker, or non-drinker or non-smoker. Those who had a drinking or smoking history were then asked for additional details. For the purpose of this study, the population was binarized into those who were current drinkers or smokers and those who were not. For salt intake status, the participants were asked to self-identify whether their eating habits were salty, and the options included four categories (light, general, salty, very salty). The subjects were also classified into two groups, salty and non-salty intake population.  Paired color retinal fundus photographs of the participants were taken using the Canon CR-2 Digital Non-Mydriatic Retinal Camera. Fundus images of this dataset are consistently sized (2736×1824 pixels). |
| Bias | 9 | Describe any efforts to address potential sources of bias | 9 | Training deep neural networks on imbalanced datasets, in which the majority of data instances belong to one class and far fewer instances belong to others, is an important problem as imbalanced datasets exist widely in the real world [28, 29]. Classifiers trained with imbalanced data are often biased towards the majority class and therefore cause higher misclassification rates for the minority class [28]. To overcome this challenge, only hypertension, hyperglycemia, and dyslipidemia and 13 related risk factors with the ratio of its two classes less than 4:1 were trained to obtain the classification model in this study **(S1 Table)**. Minority classes in each variable were oversampled using an augmentation approach until the two classes were equal. Data augmentation was conducted using Augmentor, which was an image augmentation library designed to aid the artificial generation of image data for machine learning [30]. |
| Study size | 10 | Explain how the study size was arrived at | 5 | We obtained 1222 retinal fundus images from 625 subjects from the cross-sectional study of chronic diseases dataset of Henan province in central China. |

Continued on next page

| Quantitative variables | 11 | Explain how quantitative variables were handled in the analyses. If applicable, describe which groupings were chosen and why | 23-25 | **Tables 2 and S1** |
| --- | --- | --- | --- | --- |
| Statistical methods | 12 | (*a*) Describe all statistical methods, including those used to control for confounding | 9,10 | The output of each prediction model is two continuous numbers from 0 to 1, each referring a probability of each diagnostic label, whose sum is 1. For example, in the hypertension prediction model, the results were presented as ‘hypertension: 0.897 and non-hypertension: 0.103’. The final prediction was based on the predicted labels with a higher probability, which meant that the predicted label in the example above was hypertension. For each risk factor, the accuracy of its prediction model was measured by dividing the number of correctly labeled images by the total number of images that are available in this risk factor. ROC curves were used to plot the false positive rate versus the true positive rate of the model in predicting labels of the test images. The AUC was used to evaluate the model performance for classification of each binary risk factor. |
|  |  | (*b*) Describe any methods used to examine subgroups and interactions |  |  |
|  |  | (*c*) Explain how missing data were addressed |  | N/A |
|  |  | (*d*) *Cohort study*—If applicable, explain how loss to follow-up was addressed  *Case-control study*—If applicable, explain how matching of cases and controls was addressed  *Cross-sectional study*—If applicable, describe analytical methods taking account of sampling strategy |  |  |
|  |  | (*e*) Describe any sensitivity analyses | 7,8 | The training process of transfer learning includes loading a pre-trained convolutional neural network model and its pre-trained weights, and then retraining the parameters of the fully-connected and softmax layers to classify images [21]. The pre-trained model used in this study was the Inception-v3 image recognition neural network, which was trained with a dataset of 1000 classes and more than a million images of common everyday objects from the original ImageNet database [22, 23]. Though this Inception-v3 model was not developed for medical image recognition, it has been successfully used for classifying medical images base on transfer learning methods [12, 24], which include classification of retinal fundus images [8, 14, 25]. In this study, the convolutional layers from Inception-v3 were frozen and used as fixed feature extractors. Images were first input to the Inception-v3 neural network, which extracts general features from input images and converts the image data into feature vectors. Then a classification part with fully-connected and softmax layers was trained to classify the images and outcome the predicted labels.  We trained models separately for each selected risk factors. When training each model, the whole dataset is retinal images labeled into two classes base on subjects’ corresponding risk factor outcome information and this risk factor’s classification criterion. Then, the whole dataset was randomly divided into three portions: a training dataset (80%), a tuning validation dataset (10%), and a test dataset (10%). The training and tuning validation datasets were used to develop the model, and the test dataset was used to validate the performance of the final model. During the training processes, a back propagation algorithm was used to optimize the network’s internal parameters [22], and L2 regularization technique was used to avoid overfitting [26, 27]. |
| Results | | | | |
| Participants | 13* | (a) Report numbers of individuals at each stage of study—eg numbers potentially eligible, examined for eligibility, confirmed eligible, included in the study, completing follow-up, and analysed | 5 | We obtained 1222 retinal fundus images from 625 subjects from the cross-sectional study of chronic diseases dataset of Henan province in central China. |
|  |  | (b) Give reasons for non-participation at each stage |  | N/A |
|  |  | (c) Consider use of a flow diagram |  | N/A |
| Descriptive data | 14* | (a) Give characteristics of study participants (eg demographic, clinical, social) and information on exposures and potential confounders | 21 | Table 1 |
|  |  | (b) Indicate number of participants with missing data for each variable of interest | N/A |  |
|  |  | (c) *Cohort study*—Summarise follow-up time (eg, average and total amount) | N/A |  |
| Outcome data | 15* | *Cohort study*—Report numbers of outcome events or summary measures over time | *N/A* |  |
|  |  | *Case-control study—*Report numbers in each exposure category, or summary measures of exposure | *N/A* |  |
|  |  | *Cross-sectional study—*Report numbers of outcome events or summary measures | *21-25* | Tables 1, 2 and S1 |
| Main results | 16 | (*a*) Give unadjusted estimates and, if applicable, confounder-adjusted estimates and their precision (eg, 95% confidence interval). Make clear which confounders were adjusted for and why they were included | 9 | Training deep neural networks on imbalanced datasets, in which the majority of data instances belong to one class and far fewer instances belong to others, is an important problem as imbalanced datasets exist widely in the real world [28, 29]. Classifiers trained with imbalanced data are often biased towards the majority class and therefore cause higher misclassification rates for the minority class [28]. To overcome this challenge, only hypertension, hyperglycemia, and dyslipidemia and 13 related risk factors with the ratio of its two classes less than 4:1 were trained to obtain the classification model in this study **(S1 Table)**. Minority classes in each variable were oversampled using an augmentation approach until the two classes were equal. Data augmentation was conducted using Augmentor, which was an image augmentation library designed to aid the artificial generation of image data for machine learning [30]. |
|  |  | (*b*) Report category boundaries when continuous variables were categorized | 6 | Smoking, alcohol drinking, and salt intake statuses were obtained using a questionnaire. For smoking and drinking, the participants were asked to self-identify as a current drinker (drinking more than 12 times in the past year) or smoker (having smoking habits in the past six months), former drinker or smoker, or non-drinker or non-smoker. Those who had a drinking or smoking history were then asked for additional details. For the purpose of this study, the population was binarized into those who were current drinkers or smokers and those who were not. For salt intake status, the participants were asked to self-identify whether their eating habits were salty, and the options included four categories (light, general, salty, very salty). The subjects were also classified into two groups, salty and non-salty intake population. |
|  |  | (*c*) If relevant, consider translating estimates of relative risk into absolute risk for a meaningful time period |  | N/A |

Continued on next page

| Other analyses | 17 | Report other analyses done—eg analyses of subgroups and interactions, and sensitivity analyses | 8 | We trained models separately for each selected risk factors. When training each model, the whole dataset is retinal images labeled into two classes base on subjects’ corresponding risk factor outcome information and this risk factor’s classification criterion. Then, the whole dataset was randomly divided into three portions: a training dataset (80%), a tuning validation dataset (10%), and a test dataset (10%). The training and tuning validation datasets were used to develop the model, and the test dataset was used to validate the performance of the final model. During the training processes, a back propagation algorithm was used to optimize the network’s internal parameters [22], and L2 regularization technique was used to avoid overfitting [26, 27]. |
| --- | --- | --- | --- | --- |
| Discussion | | | | |
| Key results | 18 | Summarise key results with reference to study objectives 13 |  | These results demonstrate that early microcirculatory changes may reflect the disorders of some cardiovascular risk factors before the onset of clinical cardiovascular diseases or complication eye diseases. Besides, our study is not limited to predict the above three disorders. Consistent with the study by Poplin et al. [8] in a mainly Caucasian and Hispanic population, we found that cardiovascular risk factors like age, gender, smoking status, and BMI can be predicted directly using retinal fundus images of rural population in central China. Since most of the cardiovascular risk factors can be reflected by retinal fundus images alone, our deep learning methods may, therefore, offer a novel, noninvasive measurement of early changes in the vasculature and allow the identification of people at risk of cardiovascular diseases. Importantly, our results show that applying deep learning to retinal fundus images can also predict blood erythrocyte parameters, including HCT and MCHC **(Table 2)**. Previous studies have confirmed that erythrocyte parameters are associated with cardiovascular diseases, such as metabolic syndrome [37] and that elevated blood erythrocyte parameters can have adverse effects on retinal vessel calibers [38]. |
| Limitations | 19 | Discuss limitations of the study, taking into account sources of potential bias or imprecision. Discuss both direction and magnitude of any potential bias | 14 | Despite the good performance of our models, our study has several limitations. Our dataset size is relatively small, although transfer learning algorithm can achieve a highly accurate model with a relatively small training dataset [11-13]. A larger population with more cardiovascular events would make deep learning models that could be trained and evaluated with more accuracy and higher confidence. In addition, employment of the datasets from other sources to validate our models would be beneficial for all these predictions. Overcoming these limitations using these datasets also provides an opportunity to iteratively re-train the deep learning algorithms and improve model performance. |
| Interpretation | 20 | Give a cautious overall interpretation of results considering objectives, limitations, multiplicity of analyses, results from similar studies, and other relevant evidence | 14 | In conclusion, we show that the application of deep learning to retinal fundus images is useful in the prediction of the important CVD risk factors of hypertension, dyslipidemia, diabetes. More importantly, it makes cardiovascular risk assessment of a large population both technically and economically feasible. Our work also suggests that deep learning model analysis of retinal fundus images is useful to diagnose widespread systemic vascular diseases. |
| Generalisability | 21 | Discuss the generalisability (external validity) of the study results | 12,13 | In our study, we generated a retinal fundus image dataset from a population in rural areas of central China, and demonstrate that deep learning models have the ability to predict hypertension (AUC = 0.766), hyperglycemia (AUC = 0.880), and dyslipidemia (AUC = 0.703) using retinal fundus images alone. This result achieved a higher accuracy when comparing with a recent published study by Dai et al. [36], which used a different population in China as well and showed that hypertension can be predicted using fundus images with an accuracy of 0.609. These results demonstrate that early microcirculatory changes may reflect the disorders of some cardiovascular risk factors before the onset of clinical cardiovascular diseases or complication eye diseases. Besides, our study is not limited to predict the above three disorders. |
| Other information | |  | | |
| Funding | 22 | Give the source of funding and the role of the funders for the present study and, if applicable, for the original study on which the present article is based | 15 | This research was supported by the National Key Program of Research and Development of China (2016YFC0900803; 2017YFD0400301) and and the National Natural Science Foundation of China (U1604178; U1904158). |

*Give information separately for cases and controls in case-control studies and, if applicable, for exposed and unexposed groups in cohort and cross-sectional studies.

**Note:** An Explanation and Elaboration article discusses each checklist item and gives methodological background and published examples of transparent reporting. The STROBE checklist is best used in conjunction with this article (freely available on the Web sites of PLoS Medicine at http://www.plosmedicine.org/, Annals of Internal Medicine at http://www.annals.org/, and Epidemiology at http://www.epidem.com/). Information on the STROBE Initiative is available at www.strobe-statement.org.
