# Supplementary figures and images for: Prediction of hypertension, hyperglycemia and dyslipidemia from retinal fundus photographs via deep learning: A cross-sectional study of chronic diseases in central China
Source: PLoS One. 2020 May 14;15(5):e0233166. doi: 10.1371/journal.pone.0233166 (PMC7224473; doi:10.1371/journal.pone.0233166)

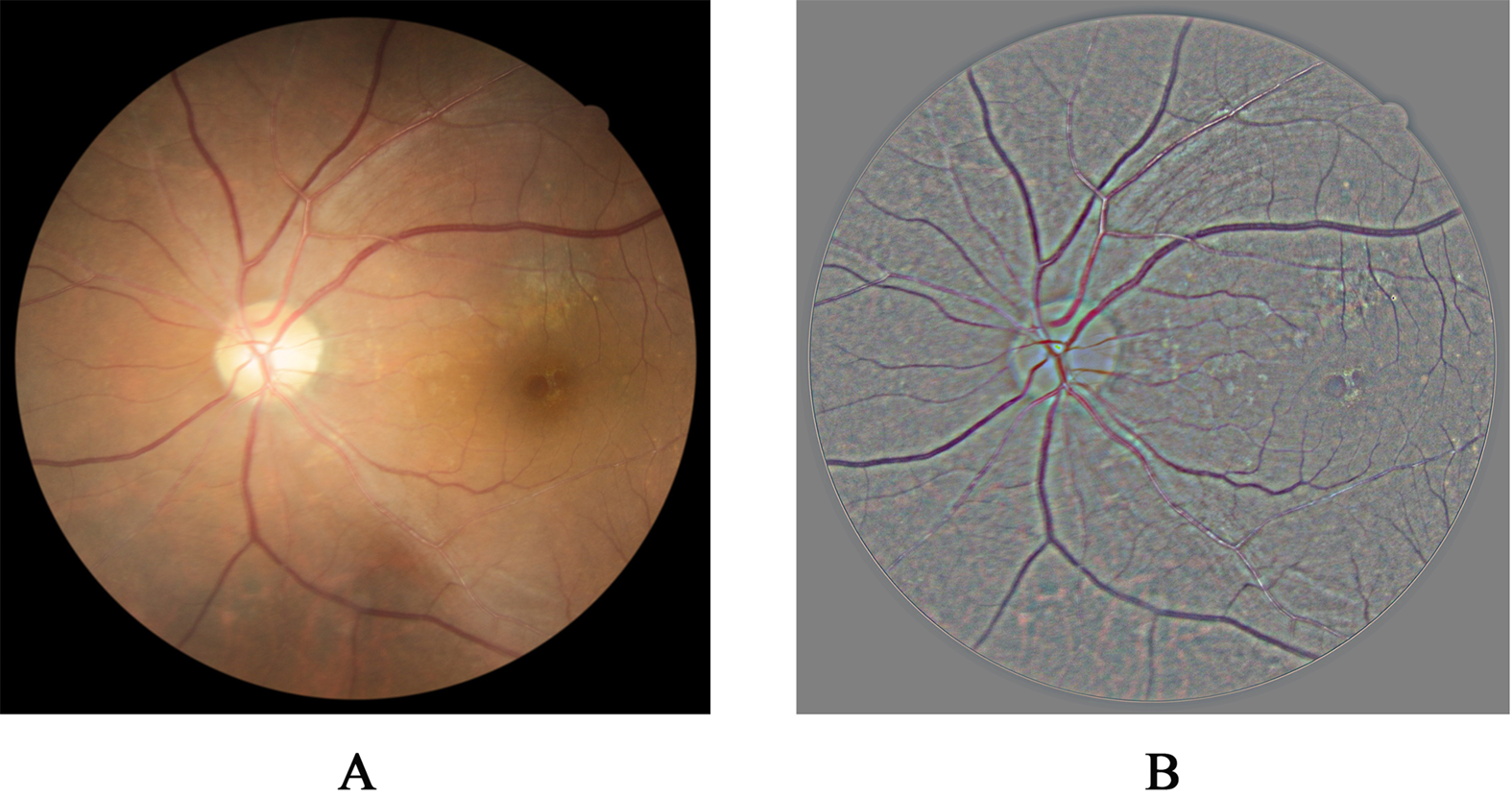

Supplement: S1 Fig — A: Original image, B: Image after pre-processing. (TIF) [file pone.0233166.s002.tif]

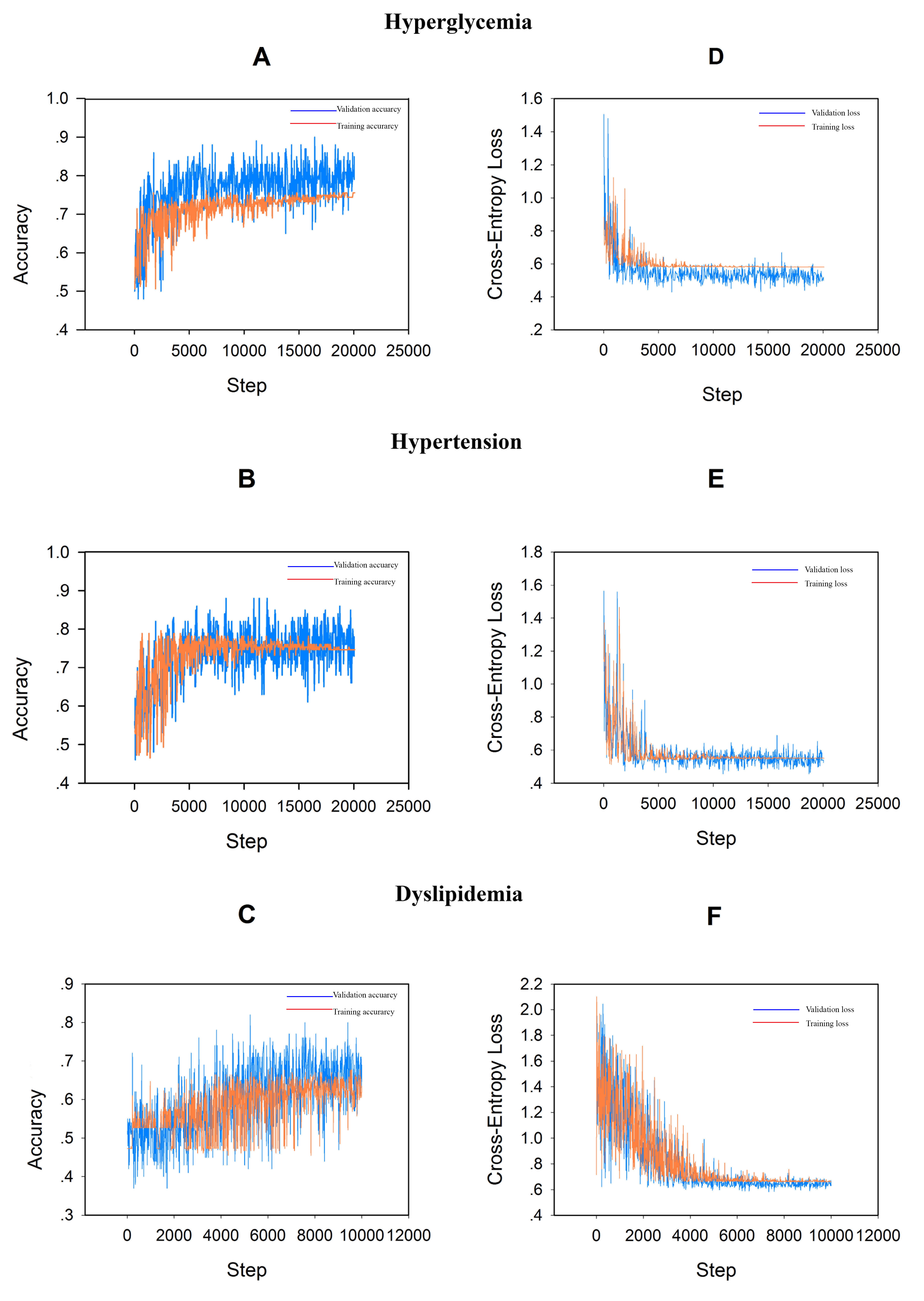

Supplement: S2 Fig — The accuracy of three disorders are shown in A (Hyperglycemia), B (Hypertension) and C (Dyslipidemia). The cross-entropy of the three disorders were shown in D-F. Training dataset: orange; Validation dataset: blue. (TIF) [file pone.0233166.s003.tif]
