# Supplementary material for: Prediction of hypertension, hyperglycemia and dyslipidemia from retinal fundus photographs via deep learning: A cross-sectional study of chronic diseases in central China
Source: PLoS One. 2020 May 14;15(5):e0233166. doi: 10.1371/journal.pone.0233166 (PMC7224473; doi:10.1371/journal.pone.0233166)
Supplement: S1 Table — (DOCX) [file pone.0233166.s004.docx]

**S1 Table. Group members of subjects and their corresponding retinal images in each variable and their image number ratio of each two groups.**

| **Labels** | **individuals** | **images** | **Ratio of images** |
| --- | --- | --- | --- |
| **D-BIL** | **624** | **1220** | **1.259** |
| Abnormal | 275 | 680 |  |
| 0-3.4 μmol/L | 349 | 540 |  |
| **Current Smoker** | **625** | **1222** | **1.406** |
| Smoker | 258 | 508 |  |
| Non-smoker | 367 | 714 |  |
| **HTN** | **625** | **1222** | **1.470** |
| SBP > 140 mmHg  or DBP > 90 mmHg | 253 | 489 |  |
| Normal | 372 | 733 |  |
| **Gender** | **624** | **1220** | **1.499** |
| Male | 248 | 485 |  |
| Female | 376 | 735 |  |
| **HCHC** | **616** | **1204** | **1.612** |
| Not low | 382 | 743 |  |
| Low HCHC | 234 | 461 |  |
| **T-Bil** | **623** | **1218** | **1.665** |
| Abnormal | 235 | 457 |  |
| 3.4-17.1 μmol/L | 388 | 761 |  |
| **Age** | **625** | **1222** | **1.704** |
| > 55 | 398 | 770 |  |
| ≤ 55 | 227 | 452 |  |
| **WHR** | **624** | **1220** | **1.766** |
| Male≥0.9, Female≥0.85 | 401 | 779 |  |
| Male<0.9, Female<0.85 | 223 | 441 |  |
| **TG** | **624** | **1220** | **1.990** |
| TG > 1.71 | 208 | 408 |  |
| TG ≤ 1.71 | 416 | 812 |  |
| **BMI** | **624** | **1220** | **2.058** |
| ≦24.0 kg/m^2^ | 203 | 399 |  |
| > 24.0 kg/m^2^ | 421 | 821 |  |
| **HCT** | **615** | **1202** | **2.386** |
| Male 40-50%, Female 35-45% | 434 | 847 |  |
| Abnormal | 181 | 355 |  |
| **Drinking** | **624** | **1220** | **2.720** |
| Drinker | 166 | 328 |  |
| Non-drinker | 458 | 892 |  |
| **FPG** | **624** | **1220** | **3.080** |
| ≤ 6.1 | 470 | 921 |  |
| > 6.1 | 154 | 299 |  |
| **Salty taste** | **624** | **1220** | **3.236** |
| lower-salt diet | 478 | 932 |  |
| High-salt diet | 146 | 288 |  |

**Abbreviation:** BMI, body mass index; D-BIL, direct bilirubin; DBP, diastolic blood pressure; FPG, fasting plasma glucose; HCT, hematocrit; MCHC, mean corpuscular hemoglobin concentration; SBP, systolic blood pressure; T-BIL, total bilirubin; TG, triglyceride; WHR, waist-hip ratio.
